# Exploring High-Dose Olanzapine for Psychosis in an Adolescent With Treatment-Resistant Schizophrenia

**DOI:** 10.7759/cureus.91355

**Published:** 2025-08-31

**Authors:** Shaurya Aggarwal, Shweta P Sahu, Puja Shah, Micah J Knobles

**Affiliations:** 1 Psychiatry and Behavioral Sciences, McGovern Medical School, University of Texas Health Science Center at Houston, Houston, USA

**Keywords:** adolescent psychosis, antipsychotic therapy, clozapine alternatives, high dose olanzapine, orally disintegrating tablets, pediatric schizophrenia, schizophrenia treatment, second generation antipsychotics, supratherapeutic dosing, treatment resistant schizophrenia

## Abstract

High-dose olanzapine has shown efficacy in treating adult patients with treatment-resistant schizophrenia, though evidence in adolescents remains limited. This report describes the case of a 17-year-old male patient with treatment-resistant schizophrenia who showed significant clinical improvement with a supratherapeutic dose of orally disintegrating olanzapine. The patient, with a 16-month history of schizophrenia and multiple hospitalizations, presented with acute psychosis and aggression after medication non-adherence. Following failed trials of paliperidone and standard-dose olanzapine, and unable to initiate clozapine due to blood draw refusal, the patient was gradually titrated to 50 mg daily of orally disintegrating olanzapine (20 mg early morning, 30 mg at bedtime). This intervention marked improvements in thought process, thought content, and paranoia, with minimal side effects. This case adds to the limited literature on high-dose olanzapine in adolescents. It suggests that supratherapeutic doses may be a viable option for treatment-resistant cases in which standard treatments are ineffective or unfeasible.

## Introduction

For the treatment of schizophrenia in children and adolescents, second-generation antipsychotic drugs are typically chosen over first-generation due to lower rates of extrapyramidal symptoms [1]. In these patients, with higher sensitivity to weight gain, lurasidone, aripiprazole, and ziprasidone are preferred, while those with agitation and aggression typically receive olanzapine or risperidone [2-4]. Aripiprazole is commonly used for the initial management of schizophrenia due to its favorable efficacy and tolerability profile, with a lower risk of weight gain, prolactin elevation, and metabolic effects [5]. If aripiprazole is ineffective, paliperidone, risperidone, and olanzapine are commonly trialed as they also have long-acting injectable formulations available [6]. Since risperidone is metabolized to paliperidone, a poor response to paliperidone may suggest risperidone would also be ineffective, though individual variations in metabolism and pharmacodynamics mean this is not absolute [7].

Following Kane's criteria, failure to respond to at least two different antipsychotics characterizes treatment-resistant schizophrenia and forms the basis for recommending clozapine in such cases [8]. Clozapine is the most effective agent for refractory schizophrenia in adolescents, and its initiation necessitates baseline and regular laboratory evaluation throughout treatment, including white blood cell count and absolute neutrophil count to monitor for agranulocytosis [9]. However, patients with severe psychosis may have reduced insight and cooperation, which can complicate adherence to laboratory protocol, particularly when they refuse blood draws.

When clozapine is not feasible due to patient refusal of required blood monitoring, olanzapine can be initiated at standard doses, with escalation to higher doses if additional improvement is needed despite partial response to standard dosing. While the evidence base for high-dose olanzapine in treatment-resistant cases is limited compared to clozapine, case reports and small studies in adults have demonstrated potential efficacy at supratherapeutic doses [10,11]. Given these clinical considerations, olanzapine represents an important treatment option when standard approaches fail.

Olanzapine is classified as a second-generation antipsychotic approved by the FDA for the treatment of schizophrenia in patients aged 13 and older, as well as for bipolar disorder, including manic or mixed episodes. It may also be used as an adjunctive treatment for treatment-resistant depression or depressive episodes associated with bipolar disorder. According to the FDA package insert, olanzapine is not approved for use in doses above 20 mg/day in adults and adolescents with schizophrenia. The safety and effectiveness of doses above 20 mg/day have not been evaluated in clinical trials [12]. However, trials in adults have shown that high-dose olanzapine (up to 60 mg/day) provides moderate to marked clinical improvements in clozapine-resistant schizophrenia without an increase in side effects [13]. Case reports show olanzapine at 50 mg/day benefits adults with treatment-resistant schizoaffective disorders [10]. Clinical trials confirm olanzapine is well tolerated at 25-45 mg/day and as effective as clozapine at 100-600 mg/day, with some patients responding only to doses above 60 mg/day [11].

A 12-week study in adolescents with schizophrenia or schizoaffective disorder (mean age 15.6 years) comparing clozapine and high-dose olanzapine found that among patients with a history of failure to respond to standard-dose olanzapine, more responded to clozapine (62%) compared to high-dose olanzapine (27%), though the difference was not statistically significant (p = .093) [14]. This highlights the potential utility of high-dose olanzapine, though its efficacy may not rival clozapine in some cases.

This case report highlights the effectiveness of high-dose orally disintegrating olanzapine tablets for treatment-resistant psychosis in an adolescent with schizophrenia who had failed to respond to other standard second-generation antipsychotics and could not access clozapine, demonstrating safety and efficacy similar to that seen in adults. It mirrors the case of an adult patient who also overcame treatment-resistant psychosis with supratherapeutic doses of the same formulation [15].

## Case presentation

A 17-year-old male patient with a history of schizophrenia and cannabis use presented with a one-day history of psychosis and aggression in the setting of medication refusal. The patient had a 16-month history of schizophrenia and seven emergency department and psychiatric hospitalizations since his first admission 15 months prior. He had a history of cannabis and psilocybin use beginning at age 16 for self-medication of anxiety symptoms, with his first psychiatric hospitalization precipitated by intentional psilocybin mushroom ingestion with suicidal intent. Both the patient and his family were unable to provide specific details about the frequency or timing of substance use, details of outside hospitalizations, or a comprehensive treatment history. Lack of access to outside psychiatric records in our electronic health system further limited available clinical information. In the outpatient setting, aripiprazole had been tried previously for an adequate period but was ineffective. His urine drug screen on current admission was negative, suggesting recent abstinence.

The patient's psychiatric deterioration was marked by progressive treatment resistance and escalating violence. Beginning with substance-induced psychosis, his condition evolved to paranoid schizophrenia with increasingly dangerous behaviors, including homicidal ideation toward family members, actively searching for and hiding knives under his mattress, and assaulting family members, store employees, and law enforcement. Family members were forced to sleep with locked doors due to fear, and he was placed on probation for assaulting a police officer. He escaped from a secure psychiatric facility and was found in a disheveled condition after being missing. This pattern demonstrated complete social and academic dysfunction, with the patient out of school for over one year and requiring increasingly intensive interventions, including restraints and seclusion.

The patient was admitted to our facility the day after discharge from a three-week hospitalization at another facility for acute psychosis and aggression, during which he was receiving oral paliperidone. His discharge medications from that previous hospitalization included paliperidone 156 mg monthly injectable for psychosis and lithium 300 mg twice daily for mood stabilization and behavioral control, as lithium had previously been beneficial according to family reports. No discrete manic episodes were documented during this hospitalization; lithium was used primarily for behavioral dysregulation associated with his psychosis, particularly aggression.

On initial evaluation, the patient was selectively mute, exhibiting echolalia and disorganized thinking. He appeared bizarre and grimacing with an overall psychotic presentation, providing ambiguous and illogical responses to questions. He was uncooperative, defensive, hostile, and suspicious, demonstrating poor insight into his condition. His presentation included paranoid delusions and bizarre beliefs, appearing internally preoccupied. During the assessment, he dramatically approached the nursing desk, reporting a nosebleed and collapsed on the floor, though no head trauma was evident on examination. The patient had a baseline BMI of 20 kg/m^2^ and a family psychiatric history significant for schizophrenia in his paternal aunt.

He was initially started on paliperidone 3 mg every night at bedtime (qhs) for psychosis, lithium ER 900 mg qhs for aggression, and guanfacine 1 mg qhs for impulsivity. The paliperidone represented continuation of treatment from his previous hospitalization, where he had received paliperidone injections and oral formulations. This was done under the hypothesis that the patient may be a rapid metabolizer, although it could not be ruled out that he was a nonresponder.

**Figure 1:**
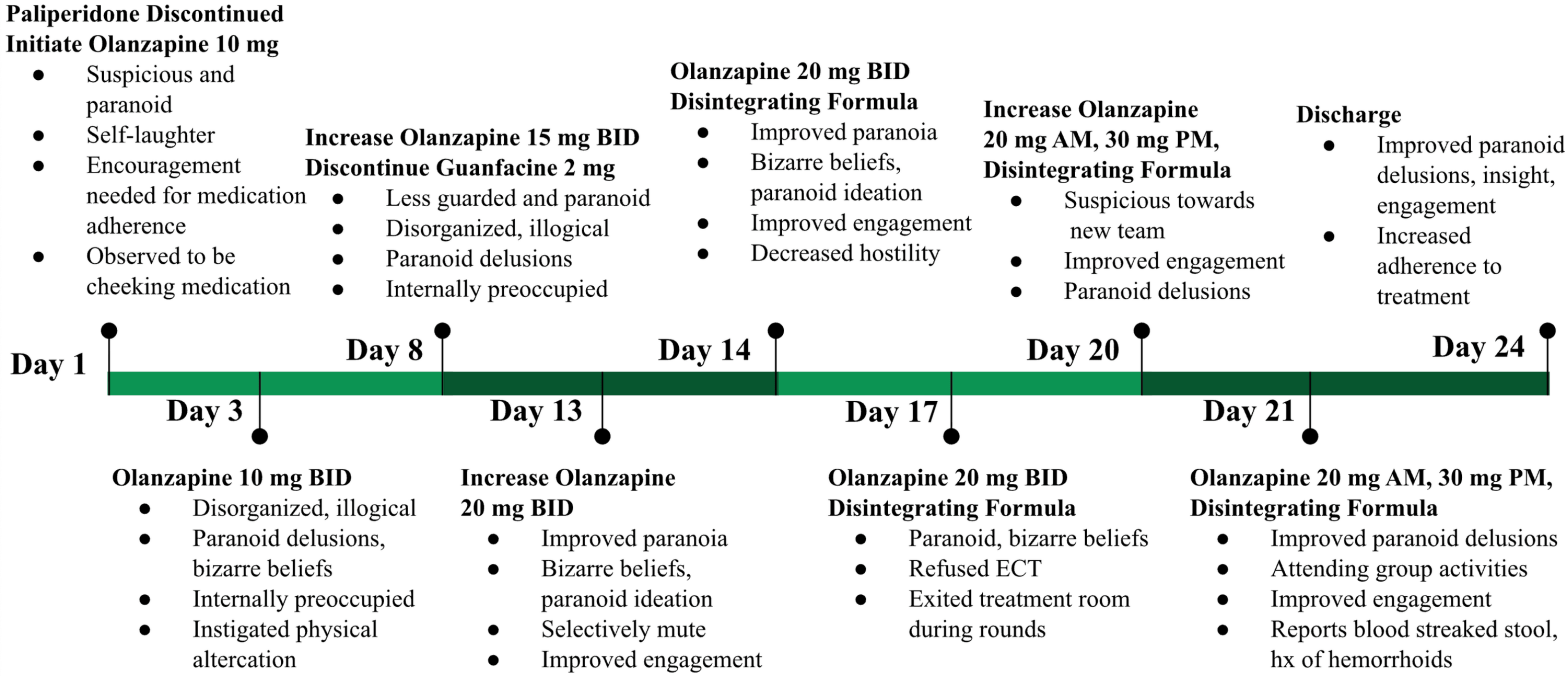
Timeline of Clinical Response During Olanzapine Titration BID: *bis in die* (twice a day)

Over the course of his five-week hospitalization, after 13 days of inadequate response to maximized oral paliperidone, he was transitioned to olanzapine (Zyprexa) 10 mg qhs (Figure 1). At this point, the patient met Kane's criteria for treatment-resistant schizophrenia, having failed adequate trials of at least six weeks each of aripiprazole in the outpatient setting and paliperidone (both long-acting injectable and oral formulations) [8]. He declined electroconvulsive therapy (ECT) for treatment-resistant psychosis and continued to refuse blood draws, preventing the collection of necessary labs, including a complete blood count (CBC), which were necessary for potentially initiating clozapine as a backup treatment.

While the patient initially appeared calmer, he continued to display bizarre behavior. Staff later discovered he was "cheeking" his medications, and without blood levels, adherence could not be verified. He was therefore switched to the orally disintegrating formulation of Zyprexa. Although some calming was noted with olanzapine, rapid titration was pursued to more effectively target his persistent psychotic symptoms in the setting of treatment-resistant schizophrenia. With gradual titration, he subsequently demonstrated progressive improvement in his symptoms.

In the week leading up to discharge, as his olanzapine orally disintegrating tablets were gradually titrated to a final supratherapeutic dose of 50 mg (20 mg early morning and 30 mg at bedtime), he continued to show noticeable improvement in thought process, thought content, and paranoia. His family observed marked improvement in rapport and paranoia during serial family therapy sessions. At discharge, he was grossly logical and organized, receptive to continuing medications on an outpatient basis, and appeared affectionate towards family. He was also amenable to blood draws, though his exact olanzapine serum concentration was inconclusive due to an incorrect sample collection method. Patient denied significant side effects other than hemorrhoids, possibly related to constipation; however, he had a prior history of hemorrhoids and constipation that resolved with polyethylene glycol. Since discharge, he has remained psychiatrically stable without further inpatient hospitalizations or emergency department visits.

## Discussion

This case contributes important insights to our understanding of high-dose olanzapine use in adolescent treatment-resistant psychosis. This adolescent patient demonstrated treatment-resistant psychosis, evidenced by failed trials of aripiprazole, paliperidone, and a standard dosage of olanzapine. Significant clinical improvement was achieved only after titrating the orally disintegrating formulation of olanzapine to a higher-than-typical dosage. Following this intervention, the patient has maintained stability without requiring further inpatient hospitalizations or emergency department visits, as documented in electronic medical records.

The successful outcome in this case suggests that careful titration to higher olanzapine doses may be a viable option for adolescents with treatment-resistant psychosis who are not candidates for clozapine. However, this must be weighed against potential risks. Notably, data from the adolescent olanzapine exposure database demonstrate that, compared with adults, adolescents experience statistically significantly greater weight gain, underscoring the need for close monitoring in this population [16]. Furthermore, evidence indicates that olanzapine-treated adolescents are more likely than adults to experience greater increases in body weight, sedation, blood lipids, serum prolactin, and liver transaminase levels [17]. In this case, the patient's baseline BMI of 20 kg/m² allowed for prioritizing acute psychosis management over immediate metabolic concerns, with plans for ongoing outpatient metabolic monitoring and interventions if needed.

When prescribing high-dose olanzapine in adolescents, treatment continuation requires careful consideration. Olanzapine is associated with a 3.32-fold increased likelihood of nonadherence or discontinuation compared to antipsychotics with lower weight gain liability [18]. While major dose-dependent patterns for weight gain are not consistently observed, olanzapine demonstrates a U-shaped discontinuation curve where doses above standard ranges may lead to increased discontinuation rates, likely due to side effects [19]. Studies have shown greater weight gain with high-dose olanzapine (≥20 mg/day) compared to clozapine (15.9 vs 3.5 lbs), requiring careful metabolic monitoring comparable to clozapine therapy [20].

Our patient's positive response indicates scientific and clinical value in further studying conventional dosing guidelines for adolescent populations. While standard doses may be sufficient for many patients, this case suggests that some adolescents with treatment-resistant schizophrenia might benefit from carefully monitored higher dosing.

## Conclusions

Current literature on high-dose olanzapine in adolescents is limited. Further study is required to establish patient selection criteria and dosing parameters in adolescent patients who may benefit from high-dose olanzapine. However, for patients in late adolescence who are physiologically approaching adulthood and respond to high-dose olanzapine, this case study supports equivalent dosing parameters, safety, and efficacy for adults and adolescents.
